# Screening for Fabry Disease Among Dialysis Patients: A Multicenter Cross-Sectional Study in Türkiye with Cascade Screening of Identified Cases

**DOI:** 10.3390/medicina62071343

**Published:** 2026-07-12

**Authors:** Kadir Gökhan Atılgan, Berrak Itır Aylı, Mehmet Deniz Aylı

**Affiliations:** 1 Division of Nephrology, Department of Internal Medicine, Ankara Etlik City Hospital, Ankara 06170, Türkiye; d_ayli@hotmail.com; 2 School of Life Sceinces, University of Westminster, London W1B 2HW, UK; itirayli@gmail.com

**Keywords:** cascade screening, chronic kidney disease, dialysis, enzyme deficiency, fabry disease, lysosomal storage disease, screening

## Abstract

*Background and Objectives:* Fabry disease (FD) is an X-linked lysosomal storage disorder caused by pathogenic *GLA* gene variants, leading to progressive multi-organ damage including end-stage renal disease. Although dialysis patients represent a high-risk population for undiagnosed FD, data from Türkiye using genetic analysis as the primary screening method remain limited. This study aimed to determine FD prevalence among hemodialysis patients across multiple centers in Türkiye and to perform cascade family screening of confirmed cases. *Materials and Methods:* This multicenter cross-sectional study screened 1359 adult hemodialysis patients across 8 centers in Ankara, Türkiye, using complete *GLA* gene sequencing. Variants were classified per American College of Medical Genetics and Genomics criteria. Patients with pathogenic variants underwent confirmatory biochemical testing (α-galactosidase A activity and plasma lyso-Gb3). Cascade screening was performed for confirmed index cases. *Results:* Among 1359 patients (mean age 62.3 ± 14.3 years; 38.5% female), *GLA* variants were identified in 12 (0.88%): 8 benign/likely benign (including 7 p.D313Y pseudodeficiency alleles), 2 unclassified variants, 1 variant of uncertain significance, and 1 confirmed classic FD (prevalence: 0.07%; 95% CI: 0.002–0.41%). Cascade screening of the index patient identified 6 carriers among 10 relatives tested (60% yield). Three of 7 carriers (43%) were initiated on enzyme replacement therapy. *Conclusions:* Among 1359 hemodialysis patients, *GLA* gene sequencing identified 12 variants (0.88%), yet only one was confirmed as a disease-causing mutation responsible for end-stage renal disease (prevalence: 0.07%). The remaining variants comprised polymorphisms, likely benign pseudodeficiency alleles and variants of uncertain significance; most of which would not have been detected by enzyme-based screening alone, as enzyme activity was normal in these carriers. Cascade screening of the single confirmed index case yielded 6 carriers among 10 relatives tested (60%), including one hemizygous male with classic FD on hemodialysis, and three carriers were initiated on enzyme replacement therapy. These findings demonstrate that *GLA* gene analysis is a valuable instrument for screening in dialysis populations, as it captures the full variant spectrum while enabling rigorous distinction between the overall *GLA* variant carrier rate and the true disease prevalence defined by variants causing end-stage renal disease. Future screening studies should report prevalence based exclusively on confirmed disease-causing variants rather than total variant counts, which have inflated prevalence estimates in prior literature.

## 1. Introduction

Fabry disease (FD; OMIM 301500) is a progressive, X-linked lysosomal storage disorder caused by pathogenic variants in the *GLA* gene (Xq22.1), leading to deficient or absent activity of the enzyme α-galactosidase A (α-Gal A) [1,2]. This enzymatic deficiency results in the progressive accumulation of globotriaosylceramide (Gb3) and its deacylated derivative, globotriaosylsphingosine (lyso-Gb3), within lysosomes of various cell types throughout the body, particularly in vascular endothelial cells, cardiomyocytes, podocytes, and neurons [1,2]. The classical phenotype in hemizygous males manifests with acroparesthesias, angiokeratomas, cornea verticillata, hypohidrosis, and gastrointestinal symptoms during childhood and adolescence, progressing to renal failure, cardiomyopathy, and cerebrovascular disease in adulthood [1,3]. Heterozygous females may exhibit a wide range of clinical manifestations, from asymptomatic to severely affected phenotypes, due to random X-chromosome inactivation [1,4]. Later-onset variants with residual enzyme activity typically present with predominant cardiac or renal involvement in adulthood [2,3].

FD is pan-ethnic, and its reported incidence based on clinical ascertainment ranges from 1 in 117,000 to 1 in 470,000 [1]. However, newborn screening studies have revealed considerably higher frequencies, reaching approximately 1 in 3100 in Italy and 1 in 1500 in Taiwan, suggesting substantial underdiagnosis [1,5,6]. The heterogeneous clinical presentation, rarity of the condition, and low physician awareness contribute to significant diagnostic delays, estimated at 10–15 years on average from symptom onset to diagnosis [3,6]. These delays are particularly concerning because disease-specific therapies, including enzyme replacement therapy (ERT) available since 2001 and pharmacological chaperone therapy (migalastat), are most effective when initiated before irreversible organ damage has occurred [1,3].

Renal involvement is one of the hallmark features of FD and a leading cause of morbidity and mortality in affected individuals. Progressive nephropathy, characterised by proteinuria, declining glomerular filtration rate, and ultimately end-stage renal disease (ESRD), occurs in the majority of classically affected males by the fourth or fifth decade of life and in a substantial proportion of females [1,7]. Given the prominent renal phenotype of FD, patients with chronic kidney disease (CKD)—particularly those on dialysis or with kidney transplants—represent a high-risk population for harbouring undiagnosed FD. This rationale has driven numerous screening studies across dialysis and transplant populations worldwide [8,9].

Screening programs in high-risk renal populations have been conducted across multiple countries and continents. In a comprehensive reanalysis of 63 screening studies spanning 1995 to 2017, Doheny et al. reported a prevalence of pathogenic *GLA* variants of 0.21% in 23,954 male dialysis patients and 0.15% in 12,866 female dialysis patients [9]. A more recent systematic review and meta-analysis by Linares et al., encompassing 55 studies with 84,062 individuals, reported an overall FD prevalence of 0.10% among dialysis patients, 0.28% among kidney transplant recipients, and 0.17% among CKD patients not on renal replacement therapy [10]. Individual screening studies have reported varying prevalence rates depending on geographic region, screening methodology, and patient selection criteria. Studies from Japan, Saudi Arabia, the United Kingdom, France, Italy, and other countries have contributed to the growing body of evidence supporting the value of systematic screening in renal populations [11,12,13,14,15,16].

In Türkiye, several screening studies have been conducted in different CKD populations. Okur et al. performed the first comprehensive screening of 1136 dialysis patients of both sexes using dried blood spots (DBS), identifying a prevalence of 0.17% [17]. Sayilar et al. screened 1527 dialysis patients in the Bursa province and reported an overall prevalence of 0.3%, with all confirmed cases being male [18]. The TURKFAB study by Turkmen et al. was the first to investigate FD prevalence among non-dialysis CKD patients (stages 1–5) in Türkiye, demonstrating a prevalence of 0.95% in 313 patients and identifying 8 additional family members through cascade screening [19]. Yenicerioglu et al. extended these findings in a multicenter study of non-dialysis CKD patients in the Aegean region; finding a prevalence of 0.2% in 1453 non-dialysis CKD patients [20]. In the transplant population, Yalin et al. conducted a large multicenter screening of 5,657 renal replacement therapy patients across Türkiye, identifying 17 *GLA* mutations, including patients with presumed primary kidney diseases [21]. Kavraz Tomar et al. further investigated FD prevalence among CKD patients across 18 sites in 8 locations in Türkiye; identifying 13 patients with pseudo mutations and four pathological results in 1904 CKD patients [22]. A recent expert consensus paper also highlighted the challenges of FD recognition and management in the Turkish context [23]. Collectively, these studies underscore the presence of undiagnosed FD in the Turkish CKD population, though the data from dialysis-specific screening with genetic confirmation remain limited.

An important aspect of FD screening is the identification of affected family members through cascade screening of index cases. Given the X-linked inheritance pattern of FD, identification of a single index patient can lead to the detection of multiple previously undiagnosed relatives, including presymptomatic individuals who may benefit from early therapeutic intervention [19,24]. The TURKFAB study demonstrated this principle effectively, as two index patients led to the identification of 8 additional family members with FD [19]. Danis et al. recently emphasized the critical role of family screening and suggested that the true prevalence of FD may be higher than currently recognized [24]. Despite these findings, the systematic integration of family screening into dialysis-based FD screening programs remains inconsistently implemented.

Although previous screening studies in Türkiye have provided valuable data, no large-scale multicenter study has simultaneously screened dialysis patients across multiple centers using genetic analysis (α-galactosidase A gene sequencing) as the primary diagnostic method and incorporated systematic family screening of identified cases. The present study aimed to determine the prevalence of FD among adult dialysis patients across 8 dialysis centers in Ankara, Türkiye using *GLA* gene analysis and to perform cascade family screening for patients diagnosed with FD. By combining population-level screening with family-based genetic investigation, this study seeks to contribute to a more accurate estimation of FD burden in the Turkish hemodialysis population and to identify presymptomatic family members who may benefit from early treatment.

The decision to employ *GLA* gene sequencing rather than enzyme activity measurement as the primary screening method was based on three considerations. First, in the laboratory performing both assays in Türkiye, the costs of full *GLA* gene sequencing and α-Gal A enzyme activity measurement were comparable, removing the traditional cost barrier to genetic-first screening. However, it should be noted that this cost equivalence is specific to the current Turkish context; it should not be assumed to apply in other healthcare settings where sequencing costs may exceed those of enzyme-based assays. Second, enzyme-based screening has well-documented limitations: dried blood spot (DBS) assays may yield incomplete results, and even serum-based enzyme measurement cannot detect carriers with normal or near-normal α-Gal A activity, particularly heterozygous females, thereby missing *GLA* variants including polymorphisms and variants of uncertain significance that may have future clinical relevance. Third, by capturing the full spectrum of GLA variants (pathogenic, VUS, and benign), this study aimed to distinguish the true Fabry disease prevalence, defined by variants causing end-stage renal disease, from the broader variant carrier rate, an important methodological distinction that has been inconsistently addressed in prior screening programs.

## 2. Materials and Methods

### 2.1. Study Design and Setting

This multicenter cross-sectional study was conducted across 8 hemodialysis centers in Ankara, Türkiye. The study protocol was approved by the Institutional Ethics Committee of Etlik City Hospital (ESH-BADEK2-2025370) and written informed consent was obtained from all participants prior to enrolment. The study was conducted in accordance with the principles of the Declaration of Helsinki.

### 2.2. Study Population and Eligibility Criteria

All adult patients (≥18 years of age) receiving maintenance hemodialysis at the 8 participating dialysis centers in Ankara who provided informed consent were considered for enrolment. The following exclusion criteria were applied: (1) histologically confirmed diagnosis of glomerulonephritis, with the exception of focal segmental glomerulosclerosis (FSGS) and minimal change disease, which were retained due to potential phenotypic overlap with Fabry nephropathy; (2) diagnosis of autosomal dominant polycystic kidney disease (ADPKD); (3) diagnosis of amyloidosis; and (4) CKD due to type 1 diabetes mellitus. All other etiologies of end-stage renal disease were included.

A total of 1474 adult hemodialysis patients were receiving maintenance hemodialysis at the 8 participating centers during the enrollment period. Of these, 34 were excluded for autosomal dominant polycystic kidney disease, 16 for type 1 diabetes mellitus, 38 for histologically confirmed glomerulonephritis, 6 for amyloidosis, and 21 patients declined participation. Exclusions for histologically confirmed glomerulonephritis and amyloidosis were applied at each center per protocol; center-level counts for these exclusion categories were not separately recorded. The final study cohort comprised 1359 patients (92.2% of the initial eligible population).

### 2.3. Data Collection

Demographic and clinical data were collected from medical records and patient interviews at the time of enrolment. Data included age, sex, dialysis vintage (duration on hemodialysis in months), and comorbidities including diabetes mellitus (DM), hypertension (HT), coronary artery disease (CAD), cerebrovascular disease (stroke), and chronic obstructive pulmonary disease (COPD).

### 2.4. Screening Strategy

The primary screening method consisted of complete *GLA* gene sequencing (full gene sequence analysis, postnatal) performed on peripheral blood samples collected in ethylenediaminetetraacetic acid (EDTA) tubes. Genetic analysis was performed using bidirectional Sanger sequencing. The analysis covered all seven coding exons and adjacent intronic regions (including exon–intron boundaries) of the *GLA* gene (NM_000169.3, OMIM 301500). Sequencing was performed in both forward and reverse directions with visual inspection of electropherograms to ensure base-call accuracy. This method detects single nucleotide variants, small insertions, and small deletions within the sequenced regions. Multiplex ligation-dependent probe amplification (MLPA) for detection of large genomic deletions, duplications, or copy number variants was not performed as part of the screening protocol. Although large *GLA* rearrangements account for a small proportion of Fabry disease-causing mutations (estimated at less than 3% of pathogenic variants), their potential contribution to undetected cases cannot be excluded and represents a methodological limitation of this study.

This approach was chosen over dried blood spot (DBS) enzyme activity screening, as genetic analysis serves as the definitive diagnostic method, particularly for detecting heterozygous female carriers who may have normal enzyme activity levels [1,24]. All identified *GLA* variants were evaluated for pathogenicity using ClinVar database classifications and published literature, American College of Medical Genetics and Genomics (ACMG) criteria, and expert consensus guidelines [25,26]. For patients found to carry pathogenic or likely pathogenic variants, confirmatory testing was performed including α-galactosidase A (α-Gal A) enzyme activity measurement in leukocytes and plasma globotriaosylsphingosine (lyso-Gb3) levels.

### 2.5. Variant Classification and Diagnosis Confirmation

Identified *GLA* gene variants were classified according to the ACMG five-tier system as pathogenic, likely pathogenic, variant of uncertain significance (VUS), likely benign, or benign [25]. A confirmed diagnosis of Fabry disease required the presence of a pathogenic or likely pathogenic *GLA* variant in combination with supporting biochemical evidence (reduced α-Gal A activity in males and/or elevated plasma lyso-Gb3) and/or clinical manifestations consistent with Fabry disease. Variants classified as benign or likely benign, including well-characterized pseudodeficiency alleles such as p.D313Y, were not considered diagnostic for Fabry disease [27,28,29,30,31,32,33].

### 2.6. Family Screening

For index patients confirmed to have Fabry disease, cascade family screening was offered to all available first- and second-degree relatives. Family members underwent *GLA* gene sequencing, and those carrying the familial variant were further evaluated with enzyme activity assays, lyso-Gb3 measurement, and comprehensive clinical assessment for Fabry-related organ involvement including echocardiography, electrocardiography, ophthalmological examination, nephrological and neurological evaluation to determine the need for disease-specific therapy.

### 2.7. Statistical Analysis

Descriptive statistics were used to summarize demographic and clinical characteristics. Continuous variables were expressed as mean ± standard deviation (SD) and categorical variables as frequencies and percentages. The prevalence of Fabry disease was calculated as the proportion of confirmed cases among the total screened population, with 95% confidence intervals (CI). Prevalence estimates were calculated with exact binomial 95% confidence intervals (Clopper–Pearson method) given the small number of events. Statistical analyses were performed using R Studio (2025.05.1) on R (4.5.1).

## 3. Results

### 3.1. Study Population Characteristics

A total of 1359 hemodialysis patients from 8 dialysis centers in Ankara, Türkiye, were enrolled and underwent *GLA* gene sequencing. The mean age was 62.28 ± 14.32 years, and 523 (38.5%) were female. The mean dialysis vintage was 73.02 ± 39.72 months. Regarding comorbidities, 501 patients (36.9%) had diabetes mellitus, 796 (58.6%) had hypertension, 359 (26.4%) had coronary artery disease, 58 (4.3%) had a history of stroke, and 83 (6.1%) had chronic obstructive pulmonary disease (Table 1).

### 3.2. GLA Gene Sequencing Results

Among the 1359 patients screened, *GLA* gene sequencing identified variants in 12 patients (0.88%), comprising 8 males and 4 females. The identified variants and their ACMG classifications are presented in Table 2.

The most frequently detected variant was p.D313Y (c.937G>T), identified in 7 patients (3 females, 4 males), classified as a likely benign pseudodeficiency allele [27,28,29,30]. The p.S126G (c.376A>G) variant in 1 heterozygous female is likewise classified as likely benign [32,33]. The intronic variant IVS4+866_867delAG was identified in two hemizygous males. This variant was previously identified in two patients in an Italian dialysis screening cohort without reduction in α-galactosidase A activity [34]. Both patients in our cohort similarly exhibited normal enzyme levels, providing concordant functional evidence across four independent cases in two countries. Furthermore, located deep within intron 4 (~866 bp from the exon–intron boundary), far from canonical splice sites, it is consistent with classification as a benign deep-intronic polymorphism [35]. Based on the deep intronic location, consistent absence of enzyme reduction, and lack of clinical Fabry manifestations in all identified carriers, this variant is most consistent with a benign polymorphism. However, as it is not yet formally classified in ClinVar and dedicated splicing functional studies have not been performed, we designate it conservatively as ‘unclassified’ rather than definitively benign, pending additional data.

One male patient carried the c.352C>T (p.Arg118Cys) variant in hemizygous state. This variant is classified as a variant of uncertain significance (VUS) in ClinVar [36]. Confirmatory biochemical testing showed normal α-galactosidase A enzyme activity, consistent with the findings of Ferreira et al. [37], who demonstrated through individual patient and family studies that p.Arg118Cys does not produce a Fabry disease phenotype. No Fabry-specific organ manifestations were identified on clinical evaluation.

One male patient carried the c.1093_1101dup(p.Ile367_Ala368insTyrThrIle) variant in hemizygous state, a likely pathogenic in-frame duplication in exon 7 [38]. Confirmatory testing revealed markedly reduced α-Gal A enzyme activity (1.4 nmol/mg/h) and significantly elevated plasma lyso-Gb3 (55.93 ng/mL), establishing a definitive diagnosis of classic Fabry disease.

The overall prevalence of confirmed Fabry disease was 0.07% (1/1359; 95% CI: 0.002–0.41%). The male-specific prevalence was 0.12% (1/836; 95% CI: 0.003–0.67%). The female-specific prevalence was 0% (0/523; 95% CI: 0–0.70%). No pathogenic *GLA* variants were identified among female hemodialysis patients in the screened cohort.

### 3.3. Clinical Characteristics of the Confirmed Fabry Disease Patient

The confirmed index patient (Patient 12, Table 2) was a 45-year-old male hemodialysis patient carrying the hemizygous c.1093_1101dup variant, a pathogenic in-frame duplication in exon 7 of the *GLA* gene. This 9-base pair duplication inserts three additional amino acids (Tyr-Thr-Ile) between positions Ile367 and Ala368, disrupting α-galactosidase A protein structure and function. The variant is classified as likely pathogenic in ClinVar [38]. His α-Gal A enzyme activity was markedly reduced at 1.4 nmol/mg/h, and plasma lyso-Gb3 was significantly elevated at 55.93 ng/mL, consistent with classic Fabry disease.

His BMI was 19.1 kg/m^2^ (height 162 cm, weight 50 kg). He did not have diabetes mellitus, hypertension, or coronary artery disease. He had been receiving maintenance hemodialysis for 25 months. Echocardiography revealed a reduced ejection fraction of 50% with interventricular septum thickness (IVS) of 1.2 cm and LVH score of 2. Electrocardiography showed ST-segment changes and left ventricular hypertrophy. The patient had NYHA Class II heart failure symptoms.

Regarding Fabry-specific manifestations, the patient exhibited cornea verticillata, acroparesthesias, angiokeratomas on physical examination, gastrointestinal symptoms, sensorineural hearing loss, and tinnitus. No unhidrosis or hypohidrosis was documented. Fabry-related edema was present on examination. He was initiated on ERT.

### 3.4. Cascade Family Screening Results

**Figure 1:**
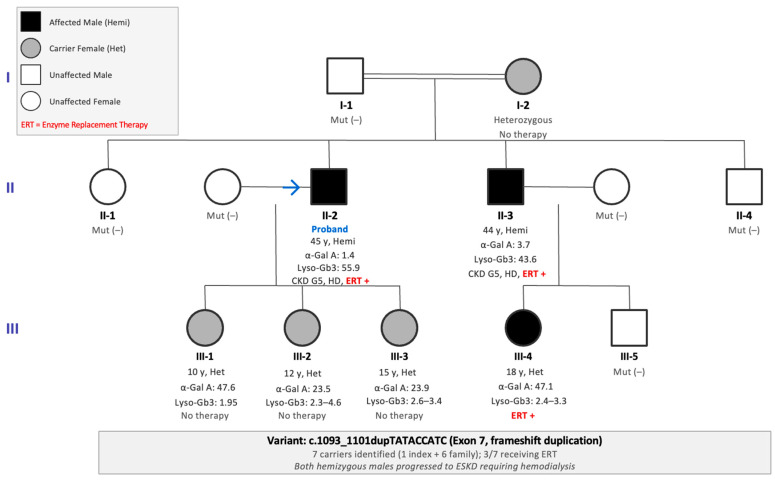
Pedigree of The Index Family. Roman numbers indicate the generation and the blue arrow shows the proband case.

Systematic cascade family screening of the index patient’s relatives identified 6 additional carriers of the c.1093_1101dup (p.Ile367_Ala368insTyrThrIle) variant from a total of 10 family members tested. The family pedigree is presented in Figure 1. The parents of the index patient were consanguineous (first cousins on the maternal side). The mother was confirmed as a heterozygous carrier and was not receiving disease-specific therapy. The father tested negative.

Among the index patient’s siblings, his brother (44 years, hemizygous) was identified with classic Fabry disease, presenting with CKD stage G5 on hemodialysis in another city. His α-Gal A activity was 3.7 nmol/mg/h with markedly elevated lyso-Gb3 of 43.6 ng/mL. Echocardiography showed an ejection fraction of 54%, IVS thickness of 1.4 cm, posterior wall thickness of 1.3 cm, and LVH score of 3. He exhibited angiokeratomas, gastrointestinal symptoms, sensorineural hearing loss, tinnitus, LVH on ECG with ST-segment changes, and NYHA Class II symptoms. He was initiated on ERT. Two other siblings (one sister, one brother) tested negative for the variant.

Three daughters of the index patient were identified as heterozygous carriers through cascade screening (Figure 1, Table 3): ages 10, 12, and 15 years. Their α-Gal A activities ranged from 23.5 to 47.6 nmol/mg/h, and lyso-Gb3 levels were mildly elevated (range: 1.95–4.6 ng/mL) with serial measurements showing stable values. Echocardiography was normal in all three (EF 60-67%, IVS 0.8–0.9 cm). None of the three daughters required disease-specific therapy at the time of evaluation; all remained under clinical follow-up.

The index patient’s brother’s daughter (18 years) was also identified as a heterozygous carrier with α-Gal A of 47.1 nmol/mg/h, lyso-Gb3 of 2.4-3.3 ng/mL, and normal echocardiography (EF 60%). In contrast to the three daughters, clinical evaluation of the brother’s daughter revealed Fabry-related manifestations including acroparesthesias, tinnitus, vertigo, and white matter hyperintensities on brain magnetic resonance imaging, indicating early neuropathic and cerebrovascular involvement. In combination with the confirmed pathogenic variant, mildly elevated plasma lyso-Gb3 (2.4–3.3 ng/mL) on serial measurements, and the severe classic Fabry disease phenotype in both hemizygous males in the family (her father and uncle, both on hemodialysis), these findings met the criteria for ERT initiation in accordance with current expert consensus guidelines recommending early treatment in heterozygous females with pathogenic variants and evidence of organ involvement [3,7]. And thus, she was initiated on ERT. The mothers of these children (wives of the index patient and his brother, respectively) tested negative for the variant, and there was no consanguinity in these marriages.

### 3.5. Summary of Screening Outcomes

**Figure 2:**
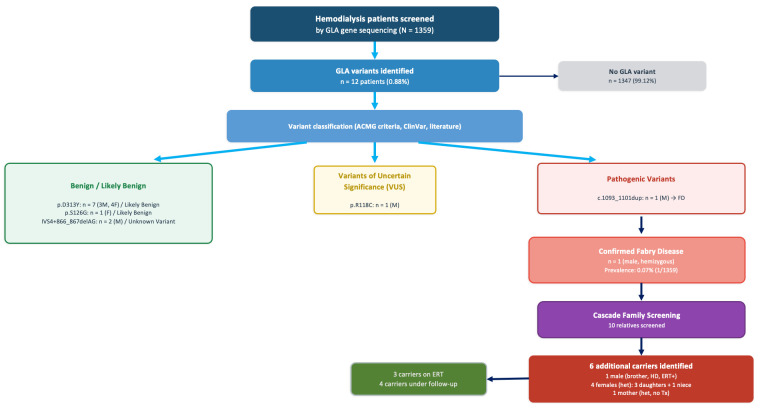
Flowchart of the Screening Results.

In summary, among 1359 hemodialysis patients screened by complete GLA gene sequencing, 12 patients (0.88%) carried *GLA* variants: 8 with benign/likely benign variants, 2 with unclassified variants, 1 patient with VUS and 1 with a confirmed pathogenic variant establishing Fabry disease (prevalence: 0.07%). Cascade family screening of the confirmed index patient identified 6 additional carriers from 10 relatives tested (60% yield), including 1 hemizygous male with classic Fabry disease requiring hemodialysis and 4 heterozygous females (3 daughters, 1 niece) plus the heterozygous mother. In total, 3 of 7 identified carriers (43%) were initiated on ERT: the two hemizygous males with classic Fabry disease and one heterozygous female with documented organ involvement demonstrating both the diagnostic and therapeutic impact of systematic cascade screening in Fabry disease (Figure 2).

## 4. Discussion

In this multicenter cross-sectional study of 1359 hemodialysis patients across 8 centers in Ankara, Türkiye, complete *GLA* gene sequencing identified one patient with confirmed classic Fabry disease, corresponding to an overall prevalence of 0.07% (male-specific: 0.12%). Cascade family screening of the index case identified 6 additional carriers among 10 relatives tested, including one hemizygous male with classic Fabry disease on hemodialysis, yielding a diagnostic rate of 60%. Three of the seven identified carriers were initiated on enzyme replacement therapy. These findings demonstrate that even in a population with a relatively low point prevalence of Fabry disease, the downstream diagnostic and therapeutic yield of systematic screening combined with cascade testing is substantial.

The confirmed prevalence of 0.07% in the present study is lower than most previously reported estimates from dialysis screening studies. In their comprehensive reanalysis of 63 screening studies, Doheny et al. reported a prevalence of pathogenic *GLA* variants of 0.21% in males and 0.15% in females [9]. More recently, Linares et al. reported an overall Fabry disease prevalence of 0.10% among dialysis patients in a meta-analysis of 55 studies encompassing 84,062 individuals [10]. Among Turkish studies, Okur et al. identified a prevalence of 0.17% among 1136 dialysis patients [17], and Sayilar et al. reported 0.3% in 1527 dialysis patients in Bursa [18]. The comparatively lower prevalence observed in our study may be attributable to several factors. First, the use of full *GLA* gene sequencing as the primary screening method, combined with rigorous variant classification according to ACMG criteria and biochemical confirmation, may have resulted in a more conservative but diagnostically precise estimate compared with studies relying on enzyme activity-based screening alone [25,26]. Second, our strict exclusion of well-characterised pseudodeficiency alleles such as p.D313Y, which accounted for more than half of the identified patients, significantly influenced the final prevalence estimate. This distinction is critical, as the inclusion or exclusion of such variants has been shown to substantially alter reported Fabry disease prevalence rates across screening programs [27,28,29,30,31,32,33]. Monda et al. demonstrated that variant reclassification using contemporary standards reduced previously reported prevalence estimates by up to 50% [31], highlighting the importance of stringent genetic interpretation in screening studies.

The predominance of p.D313Y among the identified variants merits particular attention. This variant was detected in 7 of 12 patients carrying *GLA* alterations. Although p.D313Y was historically reported as a pathogenic variant in early Fabry screening literature, subsequent biochemical and clinical studies have established that it represents a pseudodeficiency allele that causes reduced α-Gal A activity in plasma without clinically significant Gb3 accumulation or Fabry-related organ pathology [27,28,29,30]. Misclassification of such variants has led to overestimation of Fabry disease prevalence in earlier screening studies and, importantly, to unnecessary initiation of enzyme replacement therapy in some cases [31,39]. Our findings reinforce the position that screening programs must incorporate rigorous variant interpretation alongside enzymatic and biomarker assessments to avoid diagnostic inflation and inappropriate treatment. However, it must be noted that the relative costs of sequencing versus enzyme assays vary substantially across countries and healthcare systems and despite the advantages of gene sequencing, the optimal screening strategy will depend on local testing costs, laboratory infrastructure, availability of genetic counselling, and the capacity for rigorous post-screening variant classification. A formal cost-effectiveness analysis was beyond the scope of this study but would strengthen future recommendations.

Additionally, it is important to distinguish between variant detection yield and clinically actionable diagnostic yield. While gene-first screening identified 12 *GLA* variants (0.88%), only one (0.07%) proved clinically actionable; a ratio that highlights both the power and the limitation of comprehensive genetic screening. The detection of benign variants, pseudodeficiency alleles, and VUS contributes to genetic epidemiology and variant landscape characterization. However, it may also generate diagnostic uncertainty and follow-up burden: in our cohort, seven patients carrying the p.D313Y pseudodeficiency allele required additional counselling and biochemical testing to confirm the absence of disease. Screening programs employing gene-first strategies must therefore have established protocols for variant interpretation, genetic counselling, and communication of non-pathogenic results to avoid unnecessary anxiety and inappropriate treatment.

Perhaps the most clinically significant finding of this study is the high yield of cascade family screening. From a single confirmed index patient, systematic screening of 10 family members identified 6 additional carriers (60%), including a hemizygous brother with previously undiagnosed classic Fabry disease already on hemodialysis, and 4 young heterozygous females (ages 10–18 years) identified at a presymptomatic or early-symptomatic stage. This finding aligns with the TURKFAB study, in which Turkmen et al. identified 8 additional family members from two index patients through cascade screening [19], and with the observations of Danis et al., who emphasised that the true burden of Fabry disease is likely underestimated in the absence of systematic family investigation [24]. The X-linked inheritance pattern of Fabry disease makes cascade screening particularly efficient: identification of a single index case, regardless of sex, permits targeted testing of at-risk relatives across multiple generations, often revealing presymptomatic individuals who stand to benefit most from early therapeutic intervention [1,3,24].

The identification of four young heterozygous females through cascade screening is of particular clinical importance. Heterozygous females with Fabry disease have historically been regarded as “carriers” with minimal clinical manifestations; however, growing evidence demonstrates that heterozygous women experience a significant burden of disease, including renal involvement, cardiomyopathy, neuropathic pain, and reduced quality of life [4]. In our study, the three daughters and one niece of the index patient showed mildly elevated lyso-Gb3 levels with preserved cardiac and renal function, consistent with early-stage disease. The early identification of these individuals provides an opportunity for longitudinal monitoring and timely initiation of disease-specific therapy should disease progression occur, the initiation of ERT in the brother’s daughter reflects this individualised clinical judgement.

The decision to initiate ERT in the 18-year-old heterozygous niece was based on the presence of neuropathic symptoms (acroparesthesias), audiovestibular manifestations (tinnitus, vertigo), and white matter hyperintensities on brain MRI; findings consistent with early Fabry-related neuropathic and cerebrovascular involvement. These clinical manifestations, combined with an elevated lyso-Gb3 biomarker and the severe familial phenotype, fulfilled the treatment initiation criteria outlined by Wanner et al. [7] and Ortiz et al. [3], who support individualised treatment decisions in heterozygous females when evidence of organ involvement is present. Notably, the three daughters of the index patient (ages 10, 12, and 15) remained asymptomatic with normal organ function and were therefore managed with longitudinal monitoring rather than treatment initiation, illustrating the individualised approach applied across the family.

The selection of complete *GLA* gene sequencing rather than α-Gal A enzyme activity as the primary screening tool was a deliberate methodological choice that distinguishes this study from most prior dialysis screening programs. Conventional enzyme-based screening, whether by DBS or serum assay, is limited by its inability to reliably identify heterozygous female carriers due to random X-chromosome inactivation, and by its failure to detect GLA variants in individuals with residual or normal enzyme activity [1,24]. Furthermore, variant-level data provide a more complete picture of the genetic landscape in the dialysis population: of the 12 *GLA* variants identified, only one was confirmed as disease-causing after biochemical verification, whereas enzyme screening alone would have identified the single pathogenic case but would not have characterized the broader variant landscape. In the Turkish context, this distinction is particularly relevant because the cost of full GLA gene sequencing and enzyme activity measurement in the performing laboratory were comparable, eliminating the economic argument for a tiered enzyme-first strategy. These findings suggest that in settings where genetic and enzymatic testing are similarly priced, a gene-first approach may offer advantages in diagnostic yield by capturing the full variant spectrum, pathogenic, VUS, and benign, while enabling accurate distinction between true disease prevalence and the broader variant carrier rate.

In the broader Turkish context, this study contributes to the growing evidence base on Fabry disease burden in the country. While earlier studies by Okur et al. [17], Sayilar et al. [18], and the TURKFAB study by Turkmen et al. [19] have established the presence of undiagnosed Fabry disease in various Turkish CKD populations, our study is among the largest to employ full gene sequencing with systematic variant reclassification and integrated family screening in the dialysis setting. The multicenter design involving 8 dialysis units in a single metropolitan region (Ankara) provides a geographically representative yet locally focused estimate. The identification of consanguinity in the index family, the parents were first cousins on the maternal side, is a notable finding in the Turkish context, where consanguineous marriages remain prevalent in certain regions. While consanguinity does not directly alter the inheritance pattern of X-linked conditions such as Fabry disease, it may increase the probability of the pathogenic variant being present in multiple branches of the extended family through common ancestry, thereby amplifying the yield of cascade screening. In the present family, the father tested negative for the *GLA* variant, confirming that the pathogenic variant was inherited exclusively through the maternal lineage. Together with the findings of Yalin et al. [21] and Kavraz Tomar et al. [22] in broader renal replacement therapy populations, our results support the continued implementation and expansion of Fabry disease screening programs across Türkiye, with particular emphasis on integrating cascade family screening as a standard component of the diagnostic pathway.

This study has several limitations. First, the cross-sectional design limits the ability to assess long-term clinical outcomes in identified patients and carriers. Second, the study was confined to hemodialysis patients in Ankara and did not include peritoneal dialysis patients, kidney transplant recipients, or non-dialysis CKD patients, limiting generalizability to the broader CKD population. Third, the application of exclusion criteria may have led to the exclusion of patients with coexisting or misclassified Fabry disease. Fabry nephropathy can mimic or coexist with other glomerular diseases. Future screening studies should consider minimizing exclusion criteria or performing *GLA* analysis even in patients with established renal diagnoses. Fourth, while full *GLA* gene sequencing detects coding variants and splice-site mutations, it may not identify deep intronic variants, large deletions, or regulatory mutations that could cause Fabry disease. Finally, the cascade screening was limited to immediate family members, and the full extent of the disease burden within the extended family may not have been captured.

## 5. Conclusions

This multicenter study of 1359 hemodialysis patients in Türkiye identified a confirmed Fabry disease prevalence of 0.07%, with cascade family screening of the index case yielding 6 additional carriers among 10 tested relatives. The findings underscore two key principles for Fabry disease screening in dialysis populations. First, the application of rigorous variant classification and biochemical confirmation is essential to distinguish true Fabry disease from pseudodeficiency alleles and clinically non-significant variants, which together constituted the majority of *GLA* alterations identified in this cohort. Second, the high diagnostic yield of cascade family screening, which identified a previously undiagnosed hemizygous male on hemodialysis and four presymptomatic young female carriers, demonstrates that the clinical impact of screening extends well beyond the index population. These results support the integration of systematic *GLA* gene analysis and systematic cascade family screening into Fabry disease screening programs in dialysis populations.

Importantly, the use of *GLA* gene sequencing as the primary screening method enabled detection of the full variant spectrum, including polymorphisms and variants of uncertain significance, most of which would not have been detected by enzyme-based screening alone, as enzyme activity was normal in these carriers. Of the GLA variants identified, only one was confirmed to cause end-stage renal disease attributable to Fabry disease (prevalence: 0.07%), whereas previous screening studies that included polymorphisms and unverified variants in their prevalence estimates have reported substantially higher figures. This underscores the importance of basing prevalence estimates on confirmed disease-causing variants verified by biochemical and clinical criteria, rather than on the total number of *GLA* variants detected. Future screening programs should therefore consider gene-level analysis with subsequent enzyme and biomarker confirmation in settings where sequencing is accessible, affordable, and supported by expert variant interpretation and genetic counselling, and report prevalence based exclusively on variants demonstrated to cause clinically significant Fabry disease.

## Figures and Tables

**Table 1:** Demographic and Clinical Characteristics of the Study Population (N = 1359).

Characteristic	Value
Age (years), mean ± SD	62.28 ± 14.32
Female sex, n (%)	523 (38.5)
Male sex, n (%)	836 (61.5)
Dialysis vintage (months), mean ± SD	73.02 ± 39.72
**Comorbidities**	
Diabetes mellitus, n (%)	501 (36.9)
Hypertension, n (%)	796 (58.6)
Coronary artery disease, n (%)	359 (26.4)
Cerebrovascular disease (stroke), n (%)	58 (4.3)
COPD, n (%)	83 (6.1)

SD, standard deviation; COPD, chronic obstructive pulmonary disease.

**Table 2:** GLA Gene Variants Identified Among 1359 Hemodialysis Patients.

No.	Sex	Nucleotide	Protein	Zygosity	Classification	FD
1	F	c.937G>T	p.D313Y	Het	Likely benign	No
2	F	c.937G>T	p.D313Y	Het	Likely benign	No
3	M	c.937G>T	p.D313Y	Hemi	Likely benign	No
4	M	c.937G>T	p.D313Y	Hemi	Likely benign	No
5	F	c.937G>T	p.D313Y	Het	Likely benign	No
6	M	c.937G>T	p.D313Y	Hemi	Likely benign	No
7	M	c.937G>T	p.D313Y	Hemi	Likely benign	No
8	F	c.376A>G	p.S126G	Het	Likely benign	No
9	M	IVS4+866_867delAG	–	Hemi	Unknown	No
10	M	IVS4+866_867delAG	–	Hemi	Unknown	No
11	M	c.352C>T	p.R118C	Hemi	VUS	No
**12**	**M**	c.1093_1101dup	p.Ile367_Ala368insTyrThrIle	Hemi	Likely Pathogenic	Yes

F, female; M, male; Het, heterozygous; Hemi, hemizygous; VUS, variant of uncertain significance; FD, Fabry disease. Bold rows indicate pathogenic variants.

**Table 3:** Clinical and Biochemical Characteristics of Family Members Identified Through Cascade Screening.

No.	Relationship	Sex	Age	Zygosity	α-Gal A	Lyso-Gb3	EF%	IVS	CKD	ERT
Index	Proband	M	45	Hemi	1.4	55.93	50	1.2	G5/HD	Yes
F1	Brother	M	44	Hemi	3.7	43.6	54	1.4	G5/HD	Yes
F2	Mother	F	65	Het	32.3	2.6–2.8	58	1	–	No
F3	Daughter	F	10	Het	47.6	1.95	67	0.9	–	No
F4	Daughter	F	12	Het	23.5	2.3–4.6	60	0.9	–	No
F5	Daughter	F	15	Het	23.9	2.6–3.4	60	0.8	–	No
F6	Niece †	F	18	Het	47.1	2.4–3.3	60	0.8	–	Yes

α-Gal A in nmol/mg/h; Lyso-Gb3 in ng/mL; EF, ejection fraction; IVS, interventricular septum thickness (cm); CKD, chronic kidney disease stage; HD, hemodialysis; ERT, enzyme replacement therapy; Het, heterozygous; Hemi, hemizygous. † Brother’s daughter, ERT initiated based on documented acroparesthesias, tinnitus, vertigo, and white matter hyperintensities on brain MRI.

## Data Availability

The datasets used during the study have not been made publicly available due to patient privacy but are available from the corresponding author upon reasonable request.

## Abbreviations

The following abbreviations are used in this manuscript:

ACMG	American College of Medical Genetics and Genomics
ADPKD	Autosomal Dominant Polycystic Kidney Disease
BMI	Body Mass Index
CAD	Coronary Artery Disease
CI	Confidence Interval
CKD	Chronic Kidney Disease
COPD	Chronic Obstructive Pulmonary Disease
DBS	Dried Blood Spot
DM	Diabetes Mellitus
EDTA	Ethylenediaminetetraacetic Acid
EF	Ejection Fraction
ERT	Enzyme Replacement Therapy
ESRD	End-Stage Renal Disease
FD	Fabry Disease
FSGS	Focal Segmental Glomerulosclerosis
Gb3	Globotriaosylceramide
GLA	Galactosidase Alpha Gene
HD	Hemodialysis
HT	Hypertension
IVS	Interventricular Septum Thickness
LVH	Left Ventricular Hypertrophy
lyso-Gb3	Globotriaosylsphingosine
NYHA	New York Heart Association
SD	Standard Deviation
VUS	Variant of Uncertain Significance
α-Gal A	Alpha-Galactosidase A
